# Temporal Trend of Serum Perfluorooctanoic Acid and Perfluorooctane Sulfonic Acid among U.S. Adults with or without Comorbidities in NHANES 1999–2018

**DOI:** 10.3390/toxics12050314

**Published:** 2024-04-26

**Authors:** Jinhua Pan, Changping Ouyang, Shengze Zhou, Xuemei Wang, Heming Liu, Jia Zhang, Xiao Wang, Xiaoru Shi, Aimin Yang, Xiaobin Hu

**Affiliations:** 1 Institute of Epidemiology and Health Statistics, School of Public Health, Lanzhou University, Lanzhou 730000, China; panjh2017@lzu.edu.cn (J.P.); ouyangchp@163.com (C.O.); 220220913251@lzu.edu.cn (S.Z.); 220220912900@lzu.edu.cn (X.W.); 220220912581@lzu.edu.cn (H.L.); jzhang21@lzu.edu.cn (J.Z.); wangxiao21@lzu.edu.cn (X.W.); shixr2018@lzu.edu.cn (X.S.); 2 Department of Medicine and Therapeutics, The Chinese University of Hong Kong, Prince of Wales Hospital, Hong Kong 999077, China; 3 Hong Kong Institute of Diabetes and Obesity, The Chinese University of Hong Kong, Prince of Wales Hospital, Hong Kong 999077, China

**Keywords:** per- and polyfluoroalkyl substances (PFAS), serum perfluorooctanoic acid, serum perfluorooctane sulfonic acid, temporal trend, comorbidities, NHANES

## Abstract

Perfluoroalkyl and polyfluoroalkyl substances (PFAS) are associated with adverse health effects. This study examined the trend of perfluorooctanoic acid (PFOA) and perfluorooctane sulfonic acid (PFOS) levels in individuals with and without pre-existing comorbidities. We analyzed the characteristics of 13,887 participants across nine U.S. NHANES cycles (1999–2000 to 2017–2018) and calculated the geometric mean (GM) of PFOA and PFOS levels, standardized by sex and age. A joinpoint regression model was used to analyze the temporal trends of serum PFOA and PFOS levels. We observed declining PFOA and PFOS serum levels among adults in NHANES from 1999–2000 to 2017–2018. Serum PFOA and PFOS concentrations were higher in men, smokers, and individuals with pre-existing CKD, hyperlipidemia, CVD, and cancer. We observed faster decline rates in PFOA levels among individuals with diabetes and CKD and faster decline rates in PFOS levels among individuals with diabetes and those without CKD. This study provided evidence of varying levels and changing trends of PFOA and PFOS between groups with and without established chronic disease, highlighting the role of environmental chemicals in the onset and development of chronic diseases.

## 1. Introduction

Perfluoroalkyl and polyfluoroalkyl substances (PFAS) are manufactured chemicals with strong thermal and chemical stability [1,2]. They possess robust carbon–fluorine (C–F) bonds and consist of two subtypes: fully fluorinated aliphatic compounds and partially fluorinated compounds [3]. The PFAS family mainly includes perfluorooctanoic acid (PFOA), perfluorooctane sulfonic acid (PFOS), perfluorononanoic acid (PFNA), perfluorodecanoic acid (PFDA), and perfluorohexane sulfonic acid (PFHxS) [4]. Because of their unique physical and chemical properties, PFAS are widely employed in industry and commerce. They are used in the manufacturing of fire extinguishers, industrial detergents, polymerization aids, waterproof fabrics, food packaging with high temperature and oil resistance, etc. [2,3]. In addition, PFAS have bioconcentration and biomagnification effects in the food chain [5]. Polluted food and water, dust, and consumer products containing PFAS are the primary sources of PFAS exposure [6]. These substances are not easily metabolized and persist in the liver, bone, and kidney [5]. According to one systematic review, the estimated mean half-lives were 1.48 to 5.1 years for PFOA, 3.4 to 5.7 years for PFOS, and 2.84 to 8.5 years for PFHxS [7]. Due to their persistent and long half-lives, some PFAS have been listed for regulation. PFOA and PFOS are listed in the Stockholm Convention on Persistent Organic Pollutants [3].

The two perfluoroalkyls produced in the largest quantities are PFOA and PFOS, which are the most extensively researched and reported on [8]. Although the use and production of products containing PFAS have been reduced, these chemical compounds will remain in the environment for a long time due to their stability. Multiple reports have linked PFAS exposure to a variety of non-communicable diseases (NCDs). Xie et al. reported that elevated serum PFOA levels might increase the incidence of CKD (OR = 1.741) [9]. A review found evidence that PFOS was associated with testicular and kidney cancer [10]. Ward-Caviness et al. noted that PFAS exposure was significantly linked with multimorbidity (OR: 1.25, 95% CI: 1.09, 1.45) [11]. One NHANES study [12] noted a significant association of exposure to PFAS with total cholesterol and low-density lipoprotein cholesterol. A meta-analysis suggested that the risk of T2DM was linked to PFAS exposure, and this risk might increase as PFOA concentrations increase [13]. One Chinese cohort study found that PFOS exposure was significantly related to a reduced risk of chronic kidney disease (OR: 0.67) [14]. Current reports have mainly investigated the link between PFAS exposure and diseases. However, trend analysis of serum PFAS levels in individuals with and without disease is essential for protecting high-risk groups.

Blood PFOS and PFOA levels were reported to have decreased from 1999–2000 to 2017–2018 [15]. PFOA and PFOS exposure has been correlated with sociodemographic characteristics in some studies. Sonnenberg et al. found that adults, men, Asians, non-Hispanic Blacks, and non-Hispanic Whites had higher serum PFAS levels [16]. Furthermore, an NHANES study including 1325 participants found a significant association between increased PFAS exposure and elevated FT4 levels in non-smokers [17]. Although some studies have reported on the temporal trends of PFAS, few reports describe whether the decreases in serum PFOA and PFOS differ between individuals with or without disease. Therefore, our study used NHANES data to compare whether the changing trends of PFOA and PFOS blood levels differed between participants with and without disease.

## 2. Materials and Methods

### 2.1. Study Data

NHANES (https://www.cdc.gov/nchs/nhanes/, accessed on 15 April 2023) is a cross-sectional study that utilizes a complex sampling survey across the U.S. It intends to collect nationwide data on the public health and nutritional status of the population. Our study used data obtained from NHANES 1999–2000 to 2017–2018 (nine survey periods). The exclusion criteria are as follows: (1) missing blood PFOA and PFOS level data; (2) missing demographic information (sex, age, race, and smoking status); (3) missing diagnostic information on the diseases studied; and (4) age < 20 years. The study was approved by the ethics committee, and informed consent was obtained from the participants.

### 2.2. Serum PFOA and PFOS Measurement

In the NHANES data, serum samples were sent to the CDC for examination. Before being shipped to the laboratory for testing, the samples were stored at a temperature of −20 °C. PFOA and PFOS were analyzed using solid-phase extraction coupled to high-performance liquid chromatography/turbo ion spray ionization/tandem mass spectrometry with isotope-labeled internal standards [18]. The analytical measurements were strictly conducted following quality control/quality assurance guidelines. Details of serum sample storage, delivery, testing, and quality control/quality assurance are outlined in the NHANES Laboratory Procedures Manual. In NHANES 2013–2014 to 2017–2018, branched and linear isomers of PFOA and PFOS were detected. In this study, PFOA was calculated as the sum of n-perfluorooctanoate (n-PFOA) and branched perfluorooctanoate isomers (Sb-PFOA), and the concentrations of n-perfluorooctane sulfonate (n-PFOS) and perfluoromethylheptane sulfonate isomers (Sm-PFOS) were combined for total PFOS. Levels below the limits of detection (LODs) were assigned a value of LODs/√2. We summarized the LODs and detection rates of PFAS in Supplementary Table S1.

### 2.3. Sociodemographic Characteristics

The list of sociodemographic characteristics included sex (male/female), age (20–39/40–59/≥60 years), and race (White/Black/Hispanic/all other races). Additionally, we also considered cigarette use (including cigars, pipes, snuff, chewing tobacco, and electronic nicotine delivery systems) and stratified it as follows: never (never smoked more than 100 cigarettes in life), former (smoked at least 100 cigarettes in life but do not smoke at present), or current (smoked at least 100 cigarettes in life and currently smoke).

### 2.4. Definition of Variables

Participants were classified as having diabetes based on the following criteria: (1) fasting plasma glucose (FPG) ≥ 126 mg/dL (7.0 mmol/L) or (2) hemoglobin A1c ≥ 6.5% (48 mmol/mol) or (3) 2 h plasma glucose ≥ 200 mg/dL (11.1 mmol/L) during OGTT or (4) current drug therapy for T2D or (5) self-reported diabetes or sugar diabetes [19,20]. We characterized chronic kidney disease (CKD) as eGFR < 60 mL/min per 1.73 m^2^ or a urinary albumin-to-creatinine ratio ≥ 30 mg/g [21,22]. We used the CKD-EPI creatinine equation to calculate eGFR [23]. Criteria for the diagnosis of hyperlipidemia included the following: (1) triglycerides ≥ 150 mg/dL or (2) total cholesterol ≥ 200 mg/dL or (3) low-density lipoprotein (LDL) ≥ 130 mg/dL or (4) high-density lipoprotein (HDL) ≤ 40 mg/dL in men and ≤ 50 mg/dL in women or (5) participants currently using cholesterol-lowering medications [24,25]. We defined cardiovascular disease (CVD) as a self-reported history of coronary heart disease (CHD), heart failure (HF), or stroke [26,27]. Participants who self-reported as having cancer or malignancy were classified as having cancer.

### 2.5. Statistical Analysis

In this work, we outlined the characteristics of the subjects and calculated the geometric mean (GM) of serum PFOA and PFOS standardized by sex and age in each survey cycle. We also analyzed subgroup variances based on sex, smoking status, and pre-existing comorbidities. We compared group differences using the Mann–Whitney test and Kruskal–Wallis tests. Accounting for NHANES adopting complex multistage sampling, we applied modified weights calculated using the nine sampling cycles.

We used a joinpoint regression model to analyze the trend of serum PFOA and PFOS concentrations from NHANES 1999–2000 to 2017–2018. The model consists of several consecutive linear phases commonly used to characterize variations in trend data. In 1980, Lerman proposed the grid search (LGS) method [28]. LGS has become the currently used estimation method [28]. We used log-linear joinpoint regression models, and Monte Carlo permutation tests with 4499 randomly permuted datasets for analysis [29]. The average annual percentage change (AAPC) and 95% confidence intervals were used to assess the average rate of change over the entire study period, and a *t*-test was employed to determine whether the AAPC was significantly different from zero [29]. A positive value of AAPC indicates an upward trend, while a negative value indicates a downward trend [29]. In addition, we used pairwise comparison to examine whether different groups exhibited similar or different trends [30]. In the analysis, we set the significance level at 0.05. We used R version 4.2.3 and Joinpoint version 4.9.1.0 (U.S. National Cancer Institute, http://www.srab.cancer.gov/joinpoint, accessed on 28 March 2023) for the statistical analyses.

## 3. Results

### 3.1. Participants’ Characteristics

Finally, we included 13,887 participants in this analysis. The characteristics of these participants are shown in Table 1. In short, women accounted for 51.8% of the participants, Whites were the majority, participants aged 60 and older made up 34.6%, and 55.0% were former smokers. The prevalence of diabetes among participants ranged from 13.0% to 20.8% during the survey period (1999–2018), while CKD prevalence ranged from 14.2% to 27.5%, hyperlipidemia ranged from 54.5% to 80.3%, CVD ranged from 7.1% to 11.8%, and cancer ranged from 7.0% to 9.9% (Table 1).

### 3.2. Subgroup Difference

We observed subgroup differences in serum levels of PFOS and PFOA (Table 2). Serum levels of PFOS and PFOA were higher in males. Participants who smoked had higher levels of PFOS and PFOA compared to those who never smoked, and former smokers had the highest levels of PFOS and PFOA. Compared to participants without comorbidities, the median levels of PFOA were higher in participants with CKD, hyperlipidemia, CVD, and cancer by 7.7%, 16.7%, 11.1%, and 11.5%, respectively. In contrast, participants without pre-existing diabetes had higher median levels of serum PFOA. Serum PFOS concentrations were higher in individuals with diabetes, CKD, hyperlipidemia, CVD, and cancer. The median concentrations in subjects with diabetes, CKD, hyperlipidemia, CVD, and cancer were determined to be 8.3%, 13.7%, 32.5%, 38.9%, and 25.0% higher than those in participants without comorbidities, respectively (Table 2).

### 3.3. Temporal Trend

**Figure 1:**
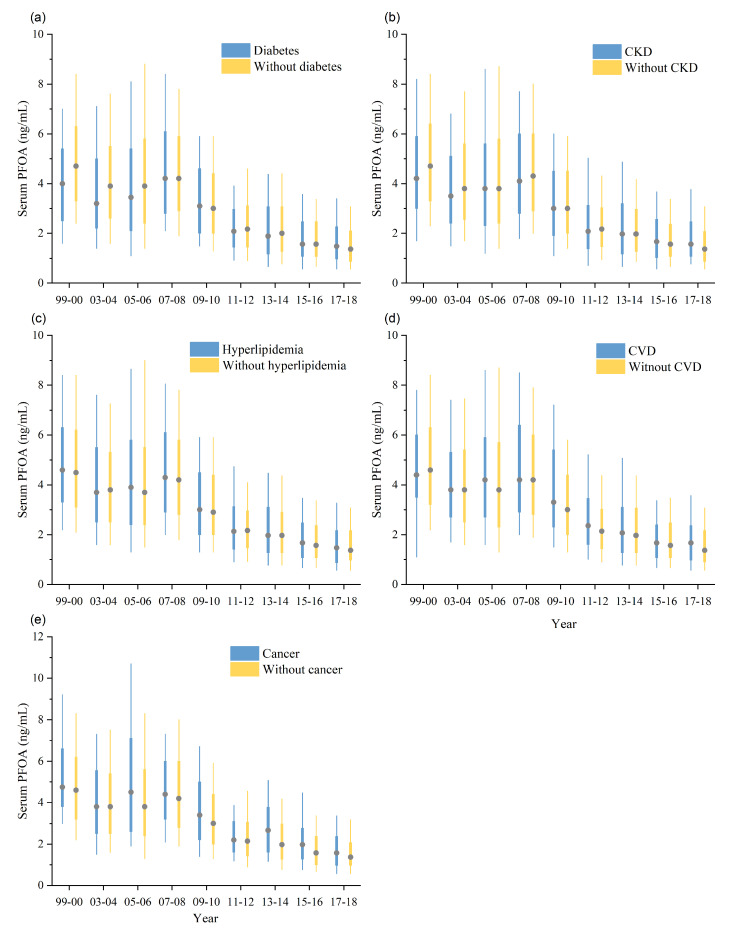
Boxplots for PFOA grouped by with and without diabetes (**a**), CKD (**b**), hyperlipidemia (**c**), CVD (**d**), and cancer (**e**) in NHANES from 1999–2000 to 2017–2018. For each box, the central mark represents the median, the edges of the box indicate the 25th and 75th percentiles and the whiskers show the 10th and 90th percentiles without considering outliers.

We found significant decreasing trends for PFOA in all subjects from 1999 to 2018 (Table 3 and Table S2). In all participants, the normalized GM of serum PFOA decreased from 4.5 ng/mL to 1.4 ng/mL between 1999–2000 and 2017–2018 (AAPC = −6.7, 95% CI: −9 to −4.3) (Table 3 and Table S2). We observed higher serum PFOA concentrations in participants with pre-existing CKD, hyperlipidemia, CVD, and cancer than in those without comorbidities, and the levels declined over the survey cycles. In contrast, participants without pre-existing diabetes had higher serum PFOA levels. Compared to PFOA levels in NHANES 1999–2000, the standardized GM levels of PFOA in NHANES 2017–2018 decreased by 68.3% (from 4.1 to 1.3 ng/mL) for diabetes (AAPC = −7.3, 95% CI: −10.4 to −4.0), by 73.2% (from 4.1 to 1.1 ng/mL) for CKD (AAPC = −6.9, 95% CI: −10.4 to −3.1), by 69.6% (from 4.6 to 1.4 ng/mL) for hyperlipidemia (AAPC = −6.3, 95% CI: −10.3 to −2.0), by 76.0% (from 5.0 to 1.2 ng/mL) for CVD (AAPC = −7.3, 95% CI: −11.3 to −3.1), and by 77.8% (from 5.4 to 1.2 ng/mL) for cancer (AAPC = −7.4, 95% CI: −10.4 to −4.3) (Figure 1, Table 3 and Table S2). According to the results of pairwise comparison, the trend in PFOA levels varied between the groups with diabetes (AAPC = −7.3) and without diabetes (AAPC = −6.4), as well as between the groups with CKD (AAPC = −6.9) and without CKD (AAPC = −6.7). Participants with diabetes and CKD showed faster decreases in PFOA levels (Table 3 and Table S4).

**Figure 2:**
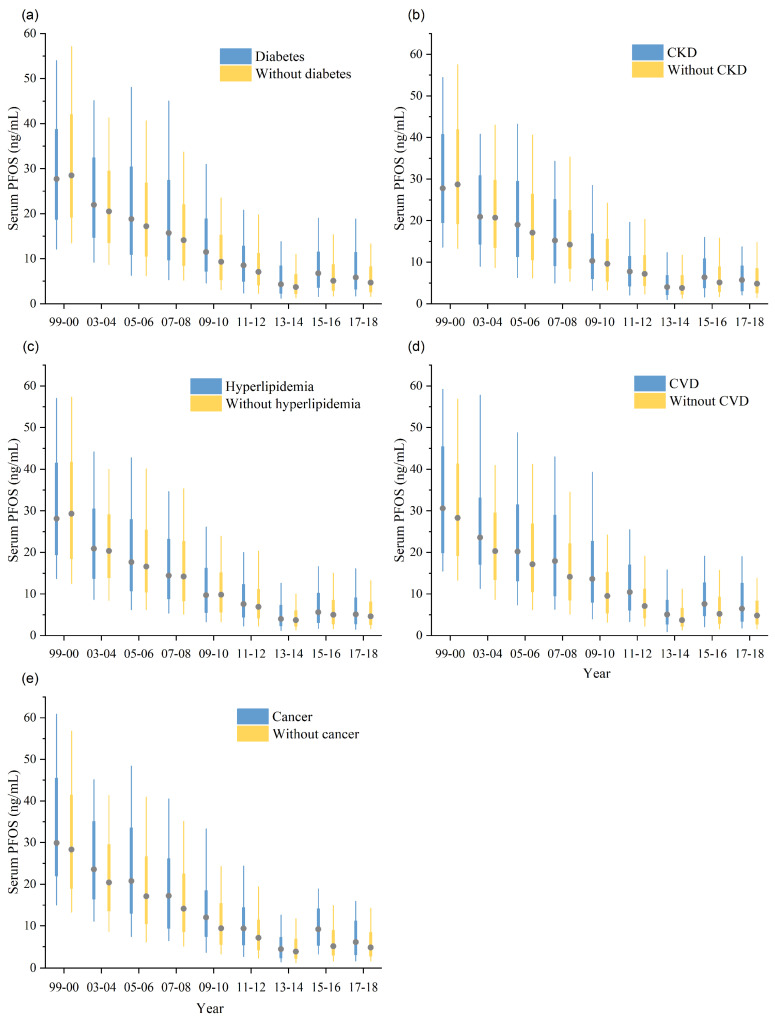
Boxplots for PFOS grouped by with and without diabetes (**a**), CKD (**b**), hyperlipidemia (**c**), CVD (**d**), and cancer (**e**) in NHANES from 1999–2000 to 2017–2018. For each box, the central mark represents the median, the edges of the box indicate the 25th and 75th percentiles and the whiskers show the 10th and 90th percentiles without considering outliers.

Similar to serum PFOA, serum PFOS levels declined among all participants in NHANES 1999–2018 (Table 3 and Table S3). Participants with comorbidities had higher serum PFOS levels. Compared to PFOS levels in NHANES 1999–2000, the standardized GM PFOS levels in NHANES 2017–2018 decreased by 83.4% (from 27.1 to 4.5 ng/mL) for diabetes (AAPC = −11.1, 95% CI: −13.5 to −8.5), decreased by 87.1% (from 26.4 to 3.4 ng/mL) for CKD (AAPC = −9.6, 95% CI: −11.2 to −7.9), decreased by 82.9% (from 28.6 to 4.9 ng/mL) for hyperlipidemia (AAPC = −8.3, 95% CI: −12.0 to −4.4), decreased by 85.6% (from 29.9 to 4.3 ng/mL) for any CVD (AAPC = −9.2, 95% CI: −11.1 to −7.3), and decreased by 84.6% (from 29.2 to 4.5 ng/mL) for cancer (AAPC = −9.8, 95% CI: −18.2 to −0.6) (Figure 2, Table 3 and Table S3). The changing trend of PFOS levels differed between groups with diabetes (AAPC = −11.1) and without diabetes (AAPC = −8.7), and between those with CKD (AAPC = −9.6) and without CKD (AAPC = −10.6). Participants with diabetes and those without CKD had faster declines in PFOS levels (Table 3 and Table S4).

## 4. Discussion

Our study compared the temporal trends of PFOA and PFOS levels in participants with and without comorbidities in US adults. The serum levels of PFOA and PFOS decreased over the survey years (NHANES 1999–2000 to 2017–2018). We found that males, smokers, and participants with CKD, hyperlipidemia, CVD, and cancer had higher serum PFOA and PFOS concentrations. In addition, we observed faster decreases in PFOA levels among individuals with diabetes and CKD, as well as faster decreases in PFOS levels among individuals with diabetes and without CKD.

Similar to the previous findings, we observed a decreasing trend in serum PFOA and PFOS concentrations from NHANES 1999–2000 to 2017–2018. In the meantime, similar results were also found in Korea, Germany, and Australia [31,32,33]. Based on measurements in 2017–2018, the standardized GMs were 1.4 ng/mL for PFOA and 4.5 ng/mL for PFOS, which were lower than Spain and higher than Germany [32,34]. Since 2000, North America and many European countries have strictly regulated the production and emissions of PFOA and PFOS [35]. In 2006, the US Environmental Protection Agency (EPA) advocated for the elimination of emissions and the use of PFOA and related substances [36]. In 2011, the Global Monitoring Plan (GMP) recommended monitoring the occurrence and changes of PFOS, PFOA, and PFHxS [37]. These measures are significant for reducing the manufacturing and emission of PFOA and PFOS.

Compared to previous reports, we observed similar results for sex differences. Men generally had higher serum concentrations of PFOS and PFOA than women. Similar reports can be found in other nations, such as Korea, Germany, Spain, and Australia [31,32,33,34]. One study conducted in Sweden [38] found a significant difference in PFAS serum levels between the sexes, showing that females had a faster metabolism rate for all PFAS compounds except PFPeS. Further stratification analysis showed that the shorter half-life in females could be related to menstruation in women of childbearing age. Reports indicated that serum PFAS can be transferred and excreted through breast milk, menstrual blood, and the umbilical cord. Serum PFOS and PFOA levels were lower (*p* < 0.01) at 3–4 months postpartum than at 2–7 weeks postpartum [39]. Serum concentrations tended to be lower in menstruating women than in women without menstrual periods, and females were likely to have higher blood PFAS levels after the climacterium [40,41]. One study noted that PFAS concentrations in maternal and cord serum accounted for 27.9% and 30.3% of the total concentrations, respectively, indicating that PFAS can be transferred to fetuses [42].

We also observed that participants who smoked had higher serum levels of PFOA and PFOS compared to those who never smoked, and former smokers had the highest levels. One American study found that former smokers had significantly higher serum PFAS levels than non-smokers [43]; similar findings were observed in a Korean report [44]. Batzella et al. estimated the half-life of blood PFOA, stratified by smoking habit, and found that the excretion rate of serum PFOA was faster in non-smokers, especially in males [45]. Specifically, the half-life of serum PFOA was 2.35 in non-smokers and 2.45 in smokers. They thought it may be associated with the different dietary habits and lifestyles of smokers and non-smokers. Currently, there is very limited literature on how smoking may affect serum PFAS concentrations.

In our study, we found that participants with pre-existing CKD, hyperlipidemia, CVD, and cancer had higher serum concentrations of PFOA and PFOS. These individuals face the health risks associated with their pre-existing conditions and the potential effects of PFAS exposure. One study identified several cytosine-guanine dinucleotide (CpG) sites related to PFAS, which were linked to gene regions associated with cancers, CVD, and renal function [46]. Xu et al. suggested that the downregulation of three microRNAs was associated with increased PFAS exposure [47]. These microRNAs were related to cardiovascular function and the growth of cancer cell lines [47]. Additionally, we observed faster declines in PFOA levels among participants with diabetes and CKD and faster declines in PFOS levels among those with diabetes and without CKD. A review found that the kidneys were the primary route of PFAS elimination, which might be related to the activity of the proximal tubules [48]. Jain et al. found that renal failure was associated with decreased renal reabsorption and greater excretion of PFOA and PFOS [5]. They observed a negative association between urinary albumin/creatinine ratios and blood levels of PFOA and PFOS. Given the association of diabetes and CKD with decreased kidney function, the studies above may partially explain our results. However, further studies are needed to explore the elimination of PFAS in individuals with pre-existing diseases.

A limitation of this study is that NHANES is a repeated cross-sectional study, which cannot explain the issue of causation, and additional longitudinal studies are needed. Secondly, considering that exposure to PFAS is a long-term, low-dose process, a single measurement is not accurate enough. This is not an association analysis and we did not consider the influence of occupation and residence on serum PFAS concentrations. Barton et al. reported that residential water district, firefighter, and military history were important determinants of serum PFAS levels. With regard to other health indicators, there were no significant differences in serum PFAS between different BMI rankings [43]. One study reported that serum PFAS levels were not statistically different between adults with overweight or obesity as compared to those within a normal weight range [2]. In addition, it was found that there was no evidence of different half-lives between BMI groups [38,45]. However, further studies are warranted to confirm these previous observations. The strengths of our study include the fact that NHANES is a large survey sample and the results are representative. Secondly, we compared the temporal trends of PFOA and PFOS concentrations among American adults with or without pre-existing comorbidities. Additionally, we calculated the GM of PFOA and PFOS standardized by sex and age.

## 5. Conclusions

We observed declining trends in serum PFOA and PFOS levels among U.S. adults in NHANES from 1999–2000 to 2017–2018. Males, smokers, and participants with pre-existing diseases have higher serum concentrations of PFOA and PFOS. PFOA levels decreased faster among individuals with diabetes and CKD, while PFOS levels declined faster among individuals with diabetes and without CKD. Our data provide evidence for future studies on the health effects of PFAS and highlight the importance of addressing the role of environmental chemicals exposure in the development of chronic diseases.

## Figures and Tables

**Table 1:** Characteristics of participants with serum PFOA and PFOS measurements in the NHANES 1999–2000 to 2017–2018.

Characteristics	1999–2000	2003–2004	2005–2006	2007–2008	2009–2010	2011–2012	2013–2014	2015-2016	2017–2018	Overall
	948 (%)	1451 (%)	1479 (%)	1738 (%)	1868 (%)	1558 (%)	1603 (%)	1634 (%)	1608 (%)	13,887 (%)
Sex										
Male	440 (46.4)	707 (48.7)	720 (48.7)	858 (49.4)	875 (46.8)	789 (50.6)	748 (46.7)	767 (46.9)	784 (48.8)	6688 (48.2)
Female	508 (53.6)	744 (51.3)	759 (51.3)	880 (50.6)	993 (53.2)	769 (49.4)	855 (53.3)	867 (53.1)	824 (51.2)	7199 (51.8)
Race										
Hispanic	371 (39.1)	330 (22.7)	331 (22.4)	500 (28.8)	542 (29)	310 (19.9)	364 (22.7)	496 (30.4)	376 (23.4)	3620 (26.1)
White	398 (42.0)	777 (53.5)	774 (52.3)	820 (47.2)	912 (48.8)	584 (37.5)	701 (43.7)	520 (31.8)	573 (35.6)	6059 (43.6)
Black	153 (16.1)	297 (20.5)	324 (21.9)	330 (19.0)	321 (17.2)	382 (24.5)	310 (19.3)	370 (22.6)	367 (22.8)	2854 (20.6)
All other races	26 (2.7)	47 (3.2)	50 (3.4)	88 (5.1)	93 (5)	282 (18.1)	228 (14.2)	248 (15.2)	292 (18.2)	1354 (9.8)
Age										
20–39	339 (35.8)	488 (33.6)	542 (36.6)	558 (32.1)	598 (32.0)	572 (36.7)	516 (32.2)	548 (33.5)	496 (30.8)	4657 (33.5)
40–59	270 (28.5)	387 (26.7)	467 (31.6)	567 (32.6)	644 (34.5)	501 (32.2)	532 (33.2)	553 (33.8)	510 (31.7)	4431 (31.9)
≥60	339 (35.8)	576 (39.7)	470 (31.8)	613 (35.3)	626 (33.5)	485 (31.1)	555 (34.6)	533 (32.6)	602 (37.4)	4799 (34.6)
Smoking status										
Never	498 (52.5)	741 (51.1)	774 (52.3)	907 (52.2)	1024 (54.8)	902 (57.9)	914 (57.0)	945 (57.8)	939 (58.4)	3435 (24.7)
Former	258 (27.2)	414 (28.5)	393 (26.6)	432 (24.9)	457 (24.5)	362 (23.2)	370 (23.1)	367 (22.5)	382 (23.8)	7644 (55.0)
Current	192 (20.3)	296 (20.4)	312 (21.1)	399 (23.0)	387 (20.7)	294 (18.9)	319 (19.9)	322 (19.7)	287 (17.8)	2808 (20.2)
DM										
No	825 (87.0)	1237 (85.3)	1267 (85.7)	1413 (81.3)	1560 (83.5)	1263 (81.1)	1285 (80.2)	1308 (80.0)	1274 (79.2)	11,432 (82.3)
Yes	123 (13.0)	214 (14.7)	212 (14.3)	325 (18.7)	308 (16.5)	295 (18.9)	318 (19.8)	326 (20.0)	334 (20.8)	2455 (17.7)
CKD										
No	734 (77.4)	1052 (72.5)	1115 (75.4)	1291 (74.3)	1447 (77.5)	1217 (78.1)	1174 (73.2)	1238 (75.8)	1380 (85.8)	10,648 (76.7)
Yes	214 (22.6)	399 (27.5)	364 (24.6)	447 (25.7)	421 (22.5)	341 (21.9)	429 (26.8)	396 (24.2)	228 (14.2)	3239 (23.3)
Hyperlipidemia										
No	187 (19.7)	390 (26.9)	429 (29.0)	688 (39.6)	763 (40.8)	666 (42.7)	658 (41.0)	743 (45.5)	719 (44.7)	5243 (37.8)
Yes	761 (80.3)	1061 (73.1)	1050 (71.0)	1050 (60.4)	1105 (59.2)	892 (57.3)	945 (59.0)	891 (54.5)	889 (55.3)	8644 (62.2)
Any CVD										
No	881 (92.9)	1280 (88.2)	1338 (90.5)	1575 (90.6)	1721 (92.1)	1448 (92.9)	1457 (90.9)	1488 (91.1)	1454 (90.4)	12,642 (91.0)
Yes	67 (7.1)	171 (11.8)	141 (9.5)	163 (9.4)	147 (7.9)	110 (7.1)	146 (9.1)	146 (8.9)	154 (9.6)	1245 (9.0)
Cancer										
No	882 (93.0)	1307 (90.1)	1344 (90.9)	1573 (90.5)	1683 (90.1)	1432 (91.9)	1445 (90.1)	1481 (90.6)	1453 (90.4)	12,600 (90.7)
Yes	66 (7.0)	144 (9.9)	135 (9.1)	165 (9.5)	185 (9.9)	126 (8.1)	158 (9.9)	153 (9.4)	155 (9.6)	1287 (9.3)

**Table 2:** The median levels of PFOA and PFOS in different groups.

**Group**	**Sample Size**	**PFOA** (**ng/mL**)		**PFOS** (**ng/mL**)	
		**Median**	***p*-Value**	**Median**	***p*-Value**
Overall	13,887	2.7	-	9.8	-
Sex			0.000		0.000
Male	6688	3.1		12.3	
Female	7199	2.3		7.8	
Smoking status			0.000		0.000
Never	7644	2.5		9.1	
Former	3435	2.9		11.9	
Now	2808	2.8		9.3	
DM			0.000		0.010
No	11,432	2.7		9.6	
Yes	2455	2.5		10.4	
CKD			0.031		0.000
No	10,648	2.6		9.5	
Yes	3239	2.8		10.8	
Hyperlipidemia			0.000		0.000
No	5243	2.4		8.3	
Yes	8644	2.8		11.0	
Any CVD			0.002		0.000
No	12,642	2.7		9.5	
Yes	1245	3.0		13.2	
Cancer			0.000		0.000
No	12,600	2.6		9.6	
Yes	1287	2.9		12.0	

**Table 3:** The average annual percent change (AAPC) and 95% CI in different groups.

Group	PFOA				PFOS			
	AAPC	95%CI	Test Statistic (t)	*p*-Value	AAPC	95%CI	Test Statistic (t)	*p*-Value
Overall	−6.7 *	(−9.0, −4.3)	−6.6	0.000	−8.8 *	(−10.8, −6.7)	−7.9	0.000
DM								
No	−6.4 *	(−8.7, −4.2)	−6.6	0.000	−8.7 *	(−12.4, −4.9)	−4.4	0.000
Yes	−7.3 *	(−10.4, −4.0)	−5.1	0.001	−11.1 *	(−13.5, −8.5)	−9.9	0.000
CKD								
No	−6.7 *	(−9.0, −4.4)	−6.6	0.000	−10.6 *	(−13.0, −8.2)	−10.0	0.000
Yes	−6.9 *	(−10.4, −3.1)	−4.3	0.004	−9.6 *	(−11.2, −7.9)	−10.9	0.000
Hyperlipidemia								
No	−5.0 *	(−9.4, −0.4)	−2.1	0.034	−9.0 *	(−10.4, −7.5)	−11.9	0.000
Yes	−6.3 *	(−10.3, −2.0)	−2.9	0.004	−8.3 *	(−12.0, −4.4)	−4.1	0.000
Any CVD								
No	−6.3 *	(−8.4, −4.2)	−5.6	0.000	−8.8 *	(−12.4, −5.0)	−4.4	0.000
Yes	−7.3 *	(−11.3, −3.1)	−3.4	0.001	−9.2 *	(−11.1, −7.3)	−9.2	0.000
Cancer								
No	−6.3 *	(−8.2, −4.3)	−6.1	0.000	−8.7 *	(−11.1, −6.4)	−7.0	0.000
Yes	−7.4 *	(−10.4, −4.3)	−4.6	0.000	−9.8 *	(−18.2, −0.6)	−2.1	0.037

* Indicates that the AAPC is significantly different from zero at the alpha = 0.05 level.

## Data Availability

The data presented in this study are openly available on the website (www.cdc.gov/nchs/nhanes/, accessed on 15 April 2023).

## Supplementary Materials

The following supporting information can be downloaded at: https://www.mdpi.com/article/10.3390/toxics12050314/s1, Table S1: Summary of limit of detection (ng/mL) and detection rates for Per- and Polyfluoroalkyl Substances; Table S2: The standardized GM of PFOA (ng/mL) in NHANES 1999–2000 to 2017–2018; Table S3: The standardized GM of PFOS (ng/mL) in NHANES 1999–2000 to 2017–2018; Table S4: Pairwise comparison of trends in serum PFOA and PFOS levels grouped by sociodemographic characteristics and comorbidities; Laboratory Quality Control Measures and Table S5: The repeat test rates of specimens in NHANES 1999–2000 to 2017–2018.
