# Supplementary material for: Temporal Trend of Serum Perfluorooctanoic Acid and Perfluorooctane Sulfonic Acid among U.S. Adults with or without Comorbidities in NHANES 1999–2018
Source: Toxics. 2024 Apr 26;12(5):314. doi: 10.3390/toxics12050314 (PMC11125848; doi:10.3390/toxics12050314)
Supplement: Supplementary file 1 [file toxics-12-00314-s001.zip › toxics-2930774-supplementary.pdf]

Supplementary Material

Temporal trend of serum perfluorooctanoic acid and perfluorooctane sulfonic acid among the U.S. adults with or without comorbidities in NHANES 1999-2018

Jinhua Pan [1], Changping Ouyang [1], Shengze Zhou [1], Xuemei Wang [1], Heming Liu [1], Jia Zhang [1], Xiao Wang [1], Xiaoru Shi [1], Aimin Yang [2,3,*] and Xiaobin Hu [1,*]

[1] Institute of Epidemiology and Health Statistics, School of Public Health, Lanzhou University, Lanzhou 730000, China; panjh2017@lzu.edu.cn (J.P.); ouyangchp@163.com (C.O.); 220220913251@lzu.edu.cn (S.Z.); 220220912900@lzu.edu.cn (X.W.); 220220912581@lzu.edu.cn (H.L.); jzhang21@lzu.edu.cn (J.Z.); wangxiao21@lzu.edu.cn (X.W.); shixr2018@lzu.edu.cn (X.S.)

[2] Department of Medicine and Therapeutics, The Chinese University of Hong Kong, Prince of Wales Hospital, Hong Kong 999077, China

[3] Hong Kong Institute of Diabetes and Obesity, The Chinese University of Hong Kong, Prince of Wales Hospital, Hong Kong 999077, China

* Correspondence: huxiaobin@lzu.edu.cn (X.H.); aiminyang@cuhk.edu.hk (A.Y.)

Supplementary Table S1. Summary of limit of detection (ng/mL) and detection rates for Per- and Polyfluoroalkyl Substances

**Chemicals**	**1999-2000**		**2003-2004**		**2005-2006**		**2007-2008**		**2009-2010**		**2011-2012**		**2013-2014**		**2015-2016**		**2017-2018**	
	**LOD**	**≥LOD(%)**	**LOD**	**≥LOD(%)**	**LOD**	**≥LOD(%)**	**LOD**	**≥LOD(%)**	**LOD**	**≥LOD(%)**	**LOD**	**≥LOD(%)**	**LOD**	**≥LOD(%)**	**LOD**	**≥LOD(%)**	**LOD**	**≥LOD(%)**
PFOA	0.1	99.9	0.1	99.6	0.1	99.6	0.1	99.9	0.1	99.8	0.1	99.5	-	-	-	-		
PFOS	0.2	100.0	0.4	99.9	0.2	99.9	0.2	99.9	0.2	99.8	0.2	99.6	-	-	-	-		
PFHxS	0.1	99.7	0.3	97.7	0.1	95.9	0.1	99.2	0.1	99.4	0.1	98.4	0.1	98.8	0.1	98.4	0.1	99.3
EPAH	0.2	92.9	0.4	3.8	0.2	6.2	0.2	3.4	0.1	5.5	0.1	5.1	-		-		-	
MPAH	0.17	97.5	0.52	24.1	0.2	82.5	0.2	69.5	0.1	75.9	0.09	53.9	0.1	44.5	0.1		0.1	59.0
PFDE	0.2	30.1	0.3	27.2	0.2	76.6	0.2	68.2	0.1	94.6	0.1	85.1	0.1	79.0	0.1	66.1	0.1	88.7
PFBS	-		0.4	0.3	0.1	7.9	0.1	0.9	0.1	0.9	0.1	1.2	0.1	0.7	-		-	
PFHP	0.4	14.1	0.3	7.6	0.4	9.5	0.4	7.6	0.1	20.5	0.1	20.1	0.1	12.5	-		-	
PFNA	0.1	95.9	0.1	98.3	0.1	99.1	0.082	99.5	0.082	99.8	0.08	99.3	0.1	98.8	0.1	98.7	0.1	92.5
PFSA	0.05	97.7	0.2	21.2	0.1	27.3	0.1	0.6	0.1	0.1	0.1	0.8	-		-		-	
PFUA	0.2	14.8	0.3	8.3	0.2	31.0	0.2	29.9	0.1	69.6	0.1	62.4	0.1	43.5	0.1	37.7	0.1	66.0
PFDO	0.2	0.8	1	0.0	0.2	2.1	0.2	2.4	0.1	5.1	0.1	9.4	0.1	16.9	0.1	2.3	-	
n-PFOA	-	-	-	-	-	-	-	-	-	-	-	-	0.1	99.1	0.1	99.2	0.1	99.6
Sb-PFOA	-	-	-	-	-	-	-	-	-	-	-	-	0.1	18.7	0.1	2.0	0.1	10.0
n-PFOS	-	-	-	-	-	-	-	-	-	-	-	-	0.1	99.0	0.1	99.4	0.1	99.7
Sm-PFOS	-	-	-	-	-	-	-	-	-	-	-	-	0.1	98.3	0.1	98.8	0.1	99.2

Supplementary Table S2. The standardized GM of PFOA (ng/mL) in NHANES 1999-2000 to 2017-2018

Group	1999-2000	2003-2004	2005-2006	2007-2008	2009-2010	2011-2012	2013-2014	2015-2016	2017-2018
Overall	4.5(4.41, 4.64)	3.6(3.51, 3.68)	3.7(3.60, 3.82)	4.1(3.99, 4.15)	3.0(2.90, 3.02)	2.1(2.03, 2.13)	1.9(1.90, 2.20)	1.6(1.56, 1.65)	1.4(1.38, 1.45)
Sex									
Male	5.0(4.84, 5.08)	4.1(3.99, 4.12)	4.4(4.37, 4.44)	4.7(4.67, 4.70)	3.4(3.39, 3.42)	2.3(2.33, 2.35)	2.2(2.20, 2.24)	1.8(1.82, 1.84)	1.6(1.59, 1.62)
Female	4.1(4.07, 4.17)	3.2(3.11, 3.23)	3.1(2.97, 3.15)	3.5(3.43, 3.56)	2.5(2.50, 2.60)	1.8(1.79, 1.89)	1.7(1.64, 1.75)	1.4(1.35, 1.44)	1.2(1.21, 1.28)
Smoke									
Never	4.5(4.26, 4.66)	3.6(3.46, 3.70)	3.6(3.46, 3.77)	4.1(3.94, 4.19)	2.9(2.85, 3.04)	2.1(2.03, 2.15)	2.0(1.89, 2.06)	1.7(1.60, 1.73)	1.4(1.40, 1.49)
Former	4.5(4.28, 4.69)	3.7(3.48, 3.86)	3.7(3.45, 3.87)	4.1(3.91, 4.22)	3.1(2.92, 3.19)	2.2(2.13, 2.34)	2.0(1.86, 2.10)	1.7(1.61, 1.74)	1.4(1.29, 1.43)
Current	4.8(4.59, 5.11)	3.5(3.31, 3.67)	4.2(3.97, 4.51)	4.3(4.13, 4.46)	3.1(2.97, 3.23)	1.9(1.81, 2.04)	1.9(1.83, 2.70)	1.5(1.36, 1.55)	1.4(1.31, 1.52)
DM									
No	4.7(4.55, 4.79)	3.7(3.59, 3.78)	3.9(3.75, 4.50)	4.1(4.02, 4.20)	3.0(2.95, 3.09)	2.2(2.12, 2.23)	2.0(1.98, 2.11)	1.7(1.63, 1.74)	1.5(1.41, 1.50)
Yes	4.1(3.77, 4.51)	3.3(3.16, 3.54)	2.8(2.54, 3.01)	4.0(3.77, 4.16)	2.8(2.67, 2.91)	1.7(1.59, 1.83)	1.7(1.59, 1.82)	1.4(1.27, 1.47)	1.3(1.20, 1.36)
CKD									
No	4.6(4.51, 4.77)	3.7(3.57, 3.78)	3.9(3.76, 4.04)	4.2(4.09, 4.28)	3.1(2.99, 3.15)	2.2(2.14, 2.26)	2.0(1.96, 2.09)	1.7(1.61, 1.72)	1.4(1.37, 1.45)
Yes	4.1(3.78, 4.41)	2.9(2.76, 3.04)	2.9(2.74, 3.13)	3.8(3.58, 3.93)	2.4(2.28, 2.61)	1.6(1.49, 1.71)	1.6(1.54, 1.74)	1.3(1.21, 1.42)	1.1(0.96, 1.23)
Hyperlipidemia									
No	4.3(4.02, 4.58)	3.4(3.26, 3.53)	3.6(3.44, 3.84)	4.1(3.92, 4.20)	2.9(2.84, 3.03)	2.1(2.00, 2.13)	2.0(1.93, 2.10)	1.6(1.58, 1.72)	1.5(1.44, 1.54)
Yes	4.6(4.44, 4.69)	3.6(3.53, 3.75)	3.7(3.57, 3.85)	4.1(3.95, 4.16)	3.0(2.89, 3.05)	2.1(2.00, 2.14)	1.9(1.83, 1.98)	1.6(1.49, 1.62)	1.4(1.30, 1.41)
Any CVD									
No	4.5(4.43, 4.67)	3.6(3.49, 3.67)	3.8(3.64, 3.87)	4.1(4.02, 4.19)	3.0(2.90, 3.03)	2.1(2.04, 2.14)	2.0(1.92, 2.04)	1.6(1.59, 1.69)	1.4(1.39, 1.46)
Yes	5.0(4.10, 5.98)	3.7(3.56, 3.94)	3.2(2.97, 3.45)	4.1(3.37, 4.74)	3.1(2.99, 3.28)	1.9(1.55, 2.19)	1.9(1.67, 2.06)	1.3(1.16, 1.44)	1.2(1.05, 1.33)
Cancer									
No	4.5(4.36, 4.60)	3.6(3.50, 3.67)	3.6(3.53, 3.76)	4.1(3.98, 4.16)	3.0(2.89, 3.02)	2.1(2.05, 2.16)	1.9(1.87, 1.98)	1.6(1.54, 1.63)	1.4(1.39, 1.47)
Yes	5.4(4.83, 5.92)	4.1(3.65, 4.56)	4.1(3.82, 4.48)	3.8(3.55, 3.99)	3.0(2.808, 3.103)	2.0(1.81, 2.22)	2.4(2.16, 2.63)	1.7(1.51, 1.81)	1.2(1.07, 1.37)

Supplementary Table S3. The standardized GM of PFOS (ng/mL) in NHANES 1999-2000 to 2017-2018

Group	1999-2000	2003-2004	2005-2006	2007-2008	2009-2010	2011-2012	2013-2014	2015-2016	2017-2018
Overall	28.7(27.98,29.36)	20.2(19.74,20.65)	17.8(17.18,18.43)	14.3(13.80,14.78)	9.9(9.54,10.26)	7.3(7.016,7.573)	4.0(3.82,4.10)	5.8(5.52,6.07)	5.0(4.81,5.28)
Sex									
Male	31.9(31.60,32.22)	22.9(22.70,23.03)	21.3(20.93,21.70)	17.7(17.40,18.00)	12.1(11.86,12.39)	8.9(8.692,9.040)	4.8(4.64,4.87)	7.3(7.10,7.52)	6.2(6.03,6.42)
Female	25.7(25.06,26.27)	17.7(17.28,18.16)	14.5(14.30,15.08)	11.1(10.78,11.47)	7.8(7.54,8.13)	5.8(5.588,6.077)	3.2(3.12,3.33)	4.4(4.16,4.61)	3.9(3.76,4.14)
Smoke									
Never	29.3(28.037,30.57)	20.7(20.00,21.43)	17.7(16.86,18.55)	15.3(14.49,16.05)	10.0(9.48,10.53)	7.5(7.11,7.82)	4.3(4.04,4.47)	6.1(5.70,6.48)	5.3(4.96,5.58)
Former	28.4(27.51,29.21)	20.2(19.06,21.24)	17.6(16.40,18.85)	13.5(12.36,14.54)	10.0(9.31,10.77)	7.7(7.06,8.29)	4.0(3.70,4.37)	5.6(5.04,6.13)	4.7(4.21,5.15)
Current	29.3(27.34,31.32)	19.6(18.77,20.43)	18.9(17.32,20.53)	14.4(13.08,15.64)	9.9(9.11,10.68)	6.1(5.48,6.76)	3.3(3.01,3.50)	5.4(4.68,6.08)	4.9(4.23,5.47)
DM									
No	29.3(28.52,30.15)	20.3(19.78,20.79)	18.4(17.65,19.06)	14.3(13.77,14.84)	9.9(9.52,10.34)	7.6(7.21,7.90)	4.1(3.92,4.27)	6.0(5.65,6.31)	5.2(4.90,5.47)
Yes	27.1(25.27,28.92)	19.7(18.71,20.74)	15.1(13.35,16.92)	14.2(12.94,15.48)	10.1(9.33,10.79)	6.5(6.00,7.05)	3.6(3.23,3.96)	5.2(4.58,5.79)	4.5(3.97,5.03)
CKD									
No	29.9(28.95,30.94)	20.9(20.31,21.52)	19.2(18.26,20.06)	14.9(14.29,15.46)	10.5(10.02,11.20)	8.0(7.64,8.41)	4.3(4.10,4.49)	6.0(5.66,6.33)	5.3(4.98,5.55)
Yes	26.4(25.22,27.56)	18.6(17.43,19.81)	15.0(13.97,16.09)	13.7(12.54,14.86)	8.5(7.94,9.02)	5.6(5.12,6.11)	3.2(2.96,3.37)	4.8(4.29,5.32)	3.4(2.89,3.92)
Hyperlipidemia									
No	28.4(26.47,30.30)	19.5(18.96,20.12)	17.8(16.69,18.95)	14.3(13.58,15.08)	10.3(9.69,10.92)	7.3(6.88,7.78)	3.9(3.75,4.06)	6.1(5.62,6.49)	5.2(4.85,5.52)
Yes	28.6(27.86,29.38)	20.4(19.79,21.02)	17.8(17.01,18.51)	14.2(13.52,14.84)	9.6(9.15,10.08)	7.2(6.85,7.61)	3.9(3.71,4.17)	5.6(5.21,5.96)	4.9(4.58,5.25)
Any CVD									
No	28.9(28.13,29.62)	20.0(19.53,20.47)	18.2(17.52,18.88)	14.4(13.88,14.89)	9.9(9.51,10.28)	7.2(6.93,7.46)	4.0(3.84,4.15)	5.9(5.59,6.20)	5.1(4.83,5.33)
Yes	29.9(26.96,32.76)	23.6(22.16,25.12)	17.0(15.31,18.62)	13.0(10.53,15.37)	10.7(9.45,11.87)	8.0(6.11,9.84)	3.9(3.15,4.56)	5.9(5.37,6.37)	4.3(3.43,5.15)
Cancer									
No	28.6(27.90,29.32)	20.1(19.62,20.59)	17.8(17.16,18.53)	14.3(13.80,14.81)	9.9(9.51,10.29)	7.3(7.04,7.63)	4.1(3.90,4.24)	5.7(5.42,5.97)	5.1(4.86,5.36)
Yes	29.2(25.08,33.31)	23.0(21.53,24.45)	17.3(15.37,19.22)	13.3(11.54,15.08)	10.2(9.13,11.35)	7.5(6.07,8.98)	3.7(3.30,4.06)	5.8(4.59,6.96)	4.5(3.79,5.30)

Supplementary Table S4. Pairwise comparison of trends in serum PFOA and PFOS levels grouped by sociodemographic characteristics and comorbidities

Cohort 1	Cohort 2	Numerator Degrees of Freedom	Denominator Degrees of Freedom	Number of Permutations	*P*-Value	Significance Level~
Serum PFOA analysis						
Diabetes	Without diabetes	4	10	4500	0.012	Rejected
Chronic kidney disease	Without chronic kidney disease	4	10	4500	0.002	Rejected
hyperlipidemia	Without hyperlipidemia	6	6	4500	0.108	Failed to reject
Cardiovascular disease	Without cardiovascular disease	4	10	4500	0.598	Failed to reject
Cancer	Without cancer	4	10	4500	0.745	Failed to reject
Serum PFOS analysis						
Diabetes	Without diabetes	6	6	4500	0.035	Rejected
Chronic kidney disease	Without chronic kidney disease	6	6	4500	0.002	Rejected
hyperlipidemia	Without hyperlipidemia	6	6	4500	0.237	Failed to reject
Cardiovascular disease	Without cardiovascular disease	6	6	4500	0.651	Failed to reject
Cancer	Without cancer	6	6	4500	0.236	Failed to reject

Laboratory Quality Control Measures

1. The NHANES quality assurance and quality control (QA/QC) protocols meet the 1988 Clinical Laboratory Improvement Act mandates.

Laboratory team performance is monitored using several techniques. NCHS and contract consultants use a structured competency assessment evaluation during visits to evaluate both the quality of the laboratory work and the quality-control procedures. Each laboratory staff member is observed for equipment operation, specimen collection and preparation; testing procedures and constructive feedback are given to each staff member. Formal retraining sessions are conducted annually to ensure that required skill levels were maintained.

2. NHANES uses several methods to monitor the quality of the analyses performed by the contract laboratories. In the MEC, these methods include performing blind split samples collected on "dry run" sessions. In addition, contract laboratories randomly perform repeat testing of all specimens. The repeat test rates were summarized in **Supplementary Table S5.**

3. NCHS developed and distributed a quality control protocol for all CDC and contract laboratories, which outlined the Westgard rules (Westgard, et al. 1981) used when running NHANES specimens. Progress reports containing any problems encountered during shipping or receipt of specimens, summary statistics for each control pool, QC graphs, instrument calibration, reagents, and any special considerations are submitted to NCHS quarterly. The reports are reviewed for trends or shifts in the data. The laboratories are required to explain any identified areas of concern.

4. All QC procedures recommended by the manufacturers were followed. Reported results for all assays meet the Division of Laboratory Science's quality control and quality assurance performance criteria for accuracy and precision, similar to specifications outlined by Westgard.

Detailed QA/QC instructions are discussed in the NHANES Laboratory Procedures Manual (LPM).

Supplementary Table S5. The repeat test rates of specimens in NHANES 1999-2000 to 2017-2018.

Survey period	1999-2000	2003-2004	2005-2006	2007-2008	2009-2010	2011-2012	2013-2014	2015-2016	2017-2018
Repeat test rate	-	2.0%	2.0%	2.0%	2.0%	2.0%	2.0%	2.0%	2.0%
